# Major-General Ireland on What Our Allies Have Done for the A. E. F.

**Published:** 1918-09

**Authors:** 


### MAJOR-GENERAL IRELAND ON WHAT OUR ALLIES HAVE DONE FOR THE A. E. F.

Major-General Ireland prefaced his introduction of Major Haven Emerson's discussion of " The Sources of War Wastage in the American Expeditionary Forces " by a brief impromptu response to the remarks of General Burtchaell, who expressed his appreciation for the services which American physicians have rendered the British armies in France. General Ireland said that he was pleased to hear from the Director General of the British armies in France that the medical officers assigned to him had proved capable and efficient, and that he considered them fortunate in having had the opportunity to serve with British medical men, because the experience which they have thus gained will be invaluable to them.

General Ireland expressed the sentiments of those who have attended the meetings of the Research Society when he further said : " All of us appreciate what we owe to our associates, the British and the French. They have taught us the methods of modern warfare. They have taught us how to take care of patients in the front line and how to get them back to the hospitals. And they have come to the meetings of the

Research Society from month to month to tell us their experiences so that we may profit from what they have learned. It will result in an enormous saving of life and effort to the American Expeditionary Forces. Any help that we may have given so far in the way of officers, nurses or men, is but a small return for these services to us and for the sacrifices they have made in those past few years in warding off the attacks of the Hun."

